# Population specificity of the *DNAI1 *gene mutation spectrum in primary ciliary dyskinesia (PCD)

**DOI:** 10.1186/1465-9921-11-174

**Published:** 2010-12-08

**Authors:** Ewa Ziętkiewicz, Barbara Nitka, Katarzyna Voelkel, Urszula Skrzypczak, Zuzanna Bukowy, Ewa Rutkiewicz, Kinga Humińska, Hanna Przystałowska, Andrzej Pogorzelski, Michał Witt

**Affiliations:** 1 Institute of Human Genetics, Poznań, Poland; 2 Institute of Tuberculosis and Lung Diseases, Rabka, Poland; 3 International Institute of Molecular and Cell Biology, Warszawa, Poland

## Abstract

**Background:**

Mutations in the *DNAI1 *gene, encoding a component of outer dynein arms of the ciliary apparatus, are the second most important genetic cause of primary ciliary dyskinesia (PCD), the genetically heterogeneous recessive disorder with the prevalence of ~1/20,000. The estimates of the *DNAI1 *involvement in PCD pathogenesis differ among the reported studies, ranging from 4% to 10%.

**Methods:**

The coding sequence of *DNAI1 *was screened (SSCP analysis and direct sequencing) in a group of PCD patients (157 families, 185 affected individuals), the first ever studied large cohort of PCD patients of Slavic origin (mostly Polish); multiplex ligation-dependent probe amplification (MLPA) analysis was performed in a subset of ~80 families.

**Results:**

Three previously reported mutations (IVS1+2-3insT, L513P and A538T) and two novel missense substitutions (C388Y and G515S) were identified in 12 families (i.e. ~8% of non-related Polish PCD patients). The structure of background SNP haplotypes indicated common origin of each of the two most frequent mutations, IVS1+2-3insT and A538T. MLPA analysis did not reveal any significant differences between patients and control samples. The Polish cohort was compared with all the previously studied PCD groups (a total of 487 families): IVS1+2-3insT remained the most prevalent pathogenetic change in *DNAI1 *(54% of the mutations identified worldwide), and the increased global prevalence of A538T (14%) was due to the contribution of the Polish cohort.

**Conclusions:**

The worldwide involvement of *DNAI1 *mutations in PCD pathogenesis in families not preselected for ODA defects ranges from 7 to 10%; this global estimate as well as the mutation profile differs in specific populations. Analysis of the background SNP haplotypes suggests that the increased frequency of chromosomes carrying A538T mutations in Polish patients may reflects local (Polish or Slavic) founder effect. Results of the MLPA analysis indicate that no large exonic deletions are involved in PCD pathogenesis.

## Background

Primary ciliary dyskinesia (PCD; MIM #242650) is a multisystem disease characterized by recurrent respiratory tract infections, sinusitis, bronchiectasis and male sub-fertility; in about half of patients it is associated with *situs inversus *(Kartagener syndrome, KS; MIM #244400), resulting from the randomization of body symmetry (for the clarity we will refer to PCD families without *s.i*. as CDO, ciliary dyskinesia only). The complex PCD phenotype is caused by the impaired motility of respiratory cilia, embryonic node cilia and sperm tails, due to ultrastructural defects of these structures [1]. Transmission electron microscopy detects various structural aberrations of the axonemal ultrastructure in over 80% of the patients [2]. The most commonly reported defects involve absence or shortening of outer (ODA) or inner (IDA) dynein arms-molecular motor complexes composed of several heavy, intermediate and light dynein chains encoded by a number of genes dispersed throughout the genome.

The prevalence of PCD is estimated at 1 in 20,000 live births (1/12,500 to 1/30,000), with the prevalence of KS being approximately two times lower [1]. PCD is usually inherited as an autosomal recessive trait, although pedigrees showing autosomal dominant or X-linked modes of inheritance have also been reported [3-6]. The complexity of the ciliary ultrastructure and the broad variety of cilia defects suggest genetic heterogeneity of the disease. Indeed, genetics of PCD is very complex, as witnessed by numerous linkage studies, which indicated several genomic regions potentially involved in PCD pathogenesis [e.g. [7-10]]; for the reviews see [11,12].

Among several genes confirmed to be directly involved in PCD pathogenesis, the major number of mutations were found in just two: *DNAI1 *(9p13.3) and *DNAH5 *(5p15.2), encoding intermediate and heavy chains of the axonemal dynein, respectively [13-21]. Mutations in other genes, coding for proteins involved in the axonemal ultrastructure (*DNAH11*, *DNAI2*, *TXNDC3*, *RSPH9*, *RSPH4A*) or assembly (*KTU*, *CRRC50*), were reported in singular PCD families only, and mutations in the *RPGR *gene were reported in rare cases of PCD associated with the X-linked retinitis pigmentosa (reviewed in [12]; see also [4,6,22-25]. Mutations in *DNAI1 *and *DNAH5*, both associated with the ODA defect phenotype, were collectively estimated to account for almost 40% (~28% and 10% for *DNAH5 *and *DNAI1*, respectively) of PCD cases [2]. Recently, other authors [20] reported much lower involvement of *DNAI1 *(4%). Here we report the results of *DNAI1 *screening performed in a large group of predominantly Polish PCD patients, the first large cohort of PCD patients of Slavic origin; the possibility that large, exonic deletions account for monoallelic mutations was also explored. Population specificity of *DNAI1 *mutation spectra is discussed in light of the SNP haplotype background of the mutations.

## Materials and methods

### Patients

A group of 157 PCD families included 185 affected individuals; parents and/or non-affected siblings were available in 115 families. Seventy-four of the families were classified as KS (if at least one affected member displayed *s.i*.); the remaining 83 were classified as CDO. At least one of the criteria listed in Table 1 had to be fulfilled to include a patient in the PCD cohort. All but six families (Czech/Slovakian) were of Polish origin. No known parental consanguinity was reported in the families (but such a possibility was not formally excluded).

**Table 1:** Clinical characteristic of the analyzed cohort

Criteria	Number of families
Typical clinical manifestation of PCD* associated with *s.i*.	74 KS

Typical clinical symptoms without *s.i.*, AND a defect in the ciliary ultrastructure in transmission electron microscope†	32 CDO^‡^

Typical clinical symptoms without *s.i.*, and the absence of ciliary motility as seen in the light microscope	51 CD

*Recurrent upper respiratory tract infections, recurrent pneumonia, chronic bronchitis, bronchiectasis, sinusitis and otitis media, reduced mucociliary clearance as shown by a negative result of a saccharine test; ^†^Usually lack of the outer/inner dynein arms, defective configuration of the microtubules; ^‡^TEM data were also available for 35 KS families. In nine families (5 CDO and 4 KS), the diagnosis was supported by low values of nasal NO ( < 100 parts per billion, ppb compared to normal > 600 ppb [37,38]).

### PCR amplification, SSCP/heteroduplex analysis and allele-specific hybridization

Genomic DNA was isolated from peripheral blood lymphocytes using a standard salting-out extraction procedure. A specific primer pair was designed for each of the 20 *DNAI1 *exons, the 5' and 3' UTR regions, and for five intronic SNPs; the length of each amplicon was < 300 bp. For the SSCP analysis, PCR-amplified segments were denatured and separated in 7 or 8% polyacrylamide (29:1) in 0.5x or 1xTBE; gels (optionally with ~2 M urea and 10% glycerol) were run at 8-10W for 20-40 h at RT or 4°C. Primer sequences, PCR conditions and detailed conditions used to separate each of the analyzed fragments are available from the authors upon request. The genotyping of SNPs and of newly found mutations was performed using dynamic ASO (allele-specific oligonucleotide) hybridization [26].

### Sequence analysis

The nucleotide changes underlying all the detected SSCP migration variants were resolved by direct sequencing of the PCR products (BigDyeTerminator v3.1 on an ABI Prism 3130XL Analyzer, Applied Biosystems); trace files were checked and edited using FinchTV1.3.1. (Geospiza Inc.). Sequences were evaluated manually using Chromas 1.45 software and FASTA sequence comparison algorithm (http://fasta.bioch.virginia.edu/fasta_www2). The reference genomic sequence was ENSG00000122735 (http://www.ensembl.org) or NG_008127.1 (http://www.ncbi.nlm.nih.gov); the exon boundaries were of the 699 aminoacid DNAI1-101 transcript ENST00000242317 (http://www.ensembl.org); the numbering of mutated nucleotide positions used throughout the text is that of cDNA.

### SNP-haplotype analysis and genetic stratification of the families

Seven intragene SNPs (rs11547035, rs4879792, rs2274591, rs3793472, rs11793196, rs9657620, rs11999046) were genotyped and parental origin of the two alleles of each SNP was determined assuming, wherever possible, no recombination among the sites. The family-based information on SNP haplotypes was used to assess the haplotype variability in all the patients. The consistency of the disease cosegregation with the haplotype variants was examined in 79 families where DNAs from proband's siblings and parents were available.

### MLPA analysis of the *DNAI1 *gene

A subset of PCD patients (~80 families) were analyzed for the potential presence of large exonic mutation(s), using commercially available kit for multiplex ligation-dependent probe amplification (MLPA) in the *DNAI1 *gene (P237-DNAI1; MRC Holland). The procedure was performed according to the manufacturer's indications (MRC Holland); briefly, hybridization of the multiple SALSA-MLPA probes (20 specific probe pairs targeting all *DNAI1 *exons) to total genomic DNA sample (50 ng per reaction) was performed at 60°C, followed by ligation at 54°C and PCR with universal, FAM-labeled MLPA primers. The resulting amplicons were separated on ABI-Prism-3130XL Analyzer; peaks were analyzed using PeakScanner v1.0 software (Applied Biosystems).

## Results

### Characteristics of the detected variants

**Figure 1:**
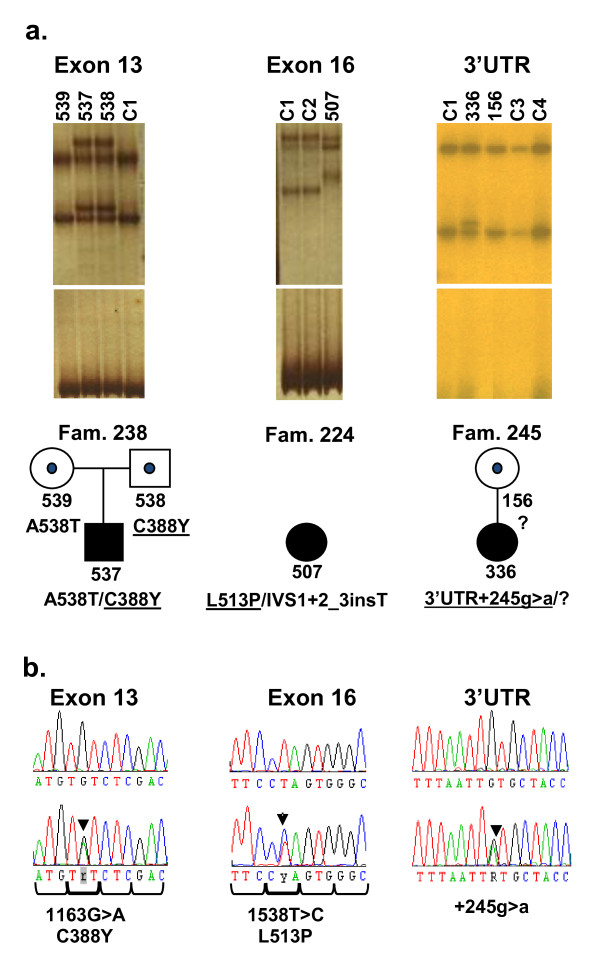
**Characteristics of three new variants detected in PCD patients**. a. Results of the SSCP analysis revealing different migration patterns, and pedigrees of the families where new mutations/SNPs were identified. New mutations (in patients 537, in his father 538, and in patients 507 and 336) are underlined. "?" denotes unknown mutation; the carrier status is indicated by a dot in the pedigree symbols. b. Chromatograms of the new sequence variants.

SSCP screening of the entire coding region of *DNAI1 *was performed in patients from 108 PCD families; systematic search for mutations was not executed in twenty-one families where the segregation of the SNP haplotype was inconsistent with that of the disease, as well as in twenty-eight families where mutations were identified in other PCD-related genes [EZ, unpublished data]. SSCP analysis revealed eight sequence variants. Two of them, in exon 1 and 11 (22G > T;A8 S and 1003G > A;V335I, respectively), were frequent SNPs (rs11547035 and rs11793196), present at high frequencies in the general population. The remaining six SSCP variants represented three previously described PCD mutations and three changes never reported before (Figure 1). The T insertion at position +2 of intron 1 (IVS1+2-3insT), the most frequent mutation described until now in PCD patients, is known to affect a donor splice site [13]. It results in retaining 132 bp of intron 1 in the mRNA and the premature termination of translation at amino acid position 25. In our study, the IVS1+2-3insT mutation was found on eleven independent chromosomes. It was homozygous in three families, accompanied by another mutation in three families and was the only mutation found in two families. Another previously reported mutation, the 1612G > A in exon 17 resulting in a missense aminoacid incorporation A538T [18], was found on eight chromosomes; it was homozygous in three families, and in two others was accompanied by IVS1+2-3insT. The third of the previously reported mutations, the 1543G > A in exon 16 (G515S) [15], was found on one chromosome in a single patient. The second chromosome of that patient carried a 1538T > C transition in exon 16 (L513P); that change was never reported before. Another new mutation, the 1163G > A transition in exon 13 (C388Y), was found in a single patient (a compound heterozygote, with IVS1+2-3insT). The third new mutation, a G > A transition 245 bp downstream from the STOP codon, was found on one PCD chromosome; no change on the second chromosome was identified in the patient.

The data from the transmission electron microscopy were available for two PCD families with the homozygous mutation A538T/A538T and for two with compound mutations IVS1+2-3insT/A538T. In all these cases, the absence of ODA and/or IDA was noted; in three of the families the absence of dynein arms was accompanied by different, non-specific defects of microtubular organization (see Table 2). Phenotype penetrance in the families with homozygous or compound mutations was consistent with the recessive mode of PCD inheritance (family members who carried only one mutated chromosome did not exhibit any clinical symptoms); the pedigrees of the families harboring the newly described mutations are presented in Figure 1.

**Table 2:** Details of patients' phenotypes and PCD-associated sequence changes detected in thirteen Polish families

Family #	Patient #	**s.i**.	nNO	% cilia with the defects identified in electron microscope	Mutation 1				Mutation 2			
					
					Exon or Intron	DNA	Protein	SVM score	Exon or Intron	DNA	Protein	SVM score
125	480	yes	na	100% ODA/IDA, 5% MT	17	1612G > A	A538T	-1.3	17	1612G > A	A538T	-1.3

106	412	yes	na	38%ODA/IDA, 46% ODAorIDA, 23% MT	17	1612G > A	A538T	-1.3	17	1612G > A	A538T	-1.3
"	413	no	na	na	"	"	"	"	"	"	"	"

151	555	yes	47	80% ODA/IDA,4% MT	17	1612G > A	A538T	-1.3	17	1612G > A	A538T	-1.3
"	556	yes	3	100% ODA/IDA	"	"	"	"	"	"	"	"

161	124	yes	na	85% ODA/IDA, 9% MT	Intr1	IVS1+2-3insT	S17fsX25	nr	17	1612G > A	A538T	-1.3

108	421	no	na	87% ODA/IDA,13% IDA	Intr1	IVS1+2-3insT	S17fsX25	nr	17	1612G > A	A538T	-1.3
"	422	no	na	100% ODA/IDA,13% IDA, 18% MT	"	"	"	"	"	"	"	"

124	478	yes	na	na	Intr1	IVS1+2-3insT	S17fsX25	nr	Intr1	IVS1+2-3insT	S17fsX25	nr

112	434	yes	na	na	Intr1	IVS1+2-3insT	S17fsX25	nr	Intr1	IVS1+2-3insT	S17fsX25	nr

231	520	no	na	na	Intr1	IVS1+2-3insT	S17fsX25	nr	Intr1	IVS1+2-3insT	S17fsX25	nr

224	507	yes	na	na	Intr1	IVS1+2-3insT	S17fsX25	nr	16	1538T > C	L513P	-2.1

238	537	yes	na	na	13	1163G > A	C388Y	-2.7	16	1543G > A	G515S	-2.4

244	548	no	48	na	Intr1	IVS1+2-3insT	S17fsX25	nr	?	?	?	?

216	355	no	37	na	Intr1	IVS1+2-3insT	S17fsX25	nr	?	?	?	?
"	356	no	49	na	"	"	"	"	"	"	"	"
"	357	no	28	na	"	"	"	"	"	"	"	"

145	336	yes	na	na	3'UTR	+245G > A	SNP	nr	?	?	?	?

None of the reported changes were found in the control group of ~200 non-PCD chromosomes. The new +245g > a in the 3'UTR, assumed to represent SNP rather than a pathogenetic mutation, was not included in the analysis of *DNAI1 *mutations prevalence. nNO-nasal NO (parts per billion); nr-not relevant; na-not available; ? - unknown; splicing D-conserved donor splice site; MT-microtubules.

**Figure 2:**
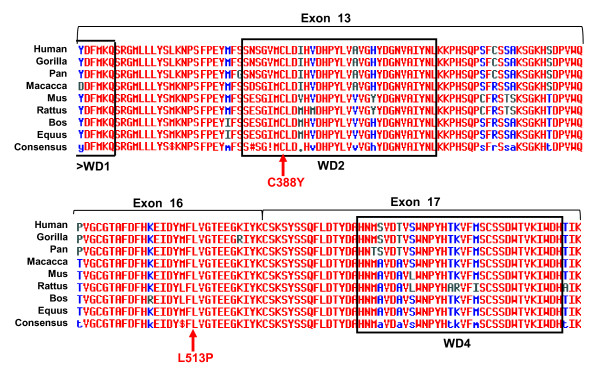
**Evolutionary conservation of the sites of two missense mutations**. The positions of two missense mutations (arrows) with respect to the position of WD-blocks 2 and 4 (boxed) in exons 13 and 16-17; species comparison indicates high evolutionary conservation.

Sequence changes that resulted in a STOP or indel mutation, or affected the two most conserved donor or acceptor consensus splice site positions, were directly assumed to represent causative PCD mutations. In case of the new missense variants (L513P and C388Y), the possibility that the change represented a non-pathological polymorphism was dismissed following a number of analyses. Interrogation of the NCBI database for human single nucleotide polymorphisms (build 131; http://www.ncbi.nlm.nih.gov/SNP) indicated that no SNPs were reported at the respective gene positions (1163G in exon 13, and 1538T in exon 16). ASO screening of the control population (~200 unrelated chromosomes from healthy Polish individuals) also did not reveal the mutated alleles. Comparison with the *DNAI1 *homologues from 9 Eutherian mammals, *P. troglodytes, P. pygmaeus, G. gorilla, M. mulatta, M. musculus, R. norvegicus, B. taurus, C. famialiris, E. caballus *(http://www.ensembl.org), indicated 100% conservation of DNA and aminoacid sequence at these two positions. This is consistent with the respective amino acids location within the DNAI1 protein: the 1163G > A substitution alters the C388 codon within the second of five highly conserved second WD-repeats (WD2) [13], and the 1538T > C in exon 16 changes the S513 codon in the highly conserved inter-repeat region, between WD3 and WD4 (Figure 2). The effect of the amino acid changes on the protein stability was examined using SNPs3 D online software http://www.snps3d.org; the SVM (Support Vector Machine) value smaller than -1.0 was assumed to indicate a deleterious effect of the amino acid substitution on the protein stability [27]. SVM scores obtained for C388Y and L513P were -2.74 and -2.17, respectively; of note, negative SVM scores (-2.50 and -1.35) were also obtained for two previously reported missense mutations, G515 S and A538T. Based on all the above observations we tentatively assumed that the newly found missense changes in exons 13 and 16 represented the disease-causing mutations.

**Figure 3:**
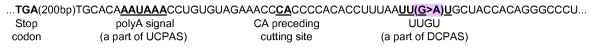
**Substitution in the *DNAI1 *3'UTR region**. The position of the substitution is highlighted; putative regulatory motifs in the *DNAI1 *3'UTR region are underlined.

The causative role of the +245G > A transition in the 3'UTR region of *DNAI1 *was less evident. This substitution was not found in the SNP database and in the analyzed control group, but comparison of the 3'UTR region in ten different species indicated low conservation of the sequence position in question. The variant was therefore analyzed in the context of sequence conservation in this region among different protein coding genes. The 3' regulatory regions are rich in regulatory elements important for the process of mRNA 3'end maturation. Although the sequence conservation and length of these motifs is not very high, some common features have been described [28]. The most important is the highly conserved polyadenylation signal, AAUAAA, a part of UCPAS (upstream core polyadenylation signal). Downstream from it is the cutting site (CS), where pre-mRNA is cut and the polyadenyl tail added; it is often preceded by a CA dinucleotide. The CS distance (10-30 bp) from two flanking segments, UCPAS and the U/GU-rich downstream core polyadenylation signal (DCPAS), is the most conserved feature of this part of the 3' regulatory region. The G > A transition found in the patient was located 32 bp downstream from the first fully conserved AAUAAA sequence after the stop codon, and within the UUGU sequence that could be a part of the DCPAS, suggesting its possible effect on mRNA polyadenylation (Figure 3). However, given the generally poor conservation of the regulatory elements among 3'UTR gene sequences, proving the importance of this mutation cannot be assessed without expression analyses. In addition, no sequence change on the complementary allele was found. The patient with the 3'UTR mutation was therefore not included in the analysis of *DNAI *mutation prevalence among PCD families.

### MLPA analysis

Direct sequencing of the whole coding sequence (exons, splice sites and UTRs), performed in two unrelated patients with monoallelic mutation (IVS1+2-3insT), did not reveal any additional sequence change. The presence of large deletions, not detectable by SSCP and/or sequencing, could explain the failure to detect the second mutated allele. To examine whether this was the case, a multiplex ligation-dependent probe amplification (MLPA) analysis of all the *DNAI1 *exons was performed in a subset of ~80 unrelated patients, including two with the monoallelic mutation and fifteen with the homozygous whole-length SNP-haplotype. The differences in the peaks' height between the samples and the control DNA from healthy individuals did not exceed 20% (not shown), indicating that no exonic deletion was present in any of the examined patients.

### Prevalence of *DNAI1 *mutations among Polish PCD families

The disease-associated changes in the *DNAI1 *sequence were found on 22 non-related chromosomes from twelve families (including two with the monoallelic mutation), which accounts for 8% of the analyzed cohort of 157 PCD families. Interestingly, when CDO and KS families were considered separately, the proportion of those with *DNAI *mutations was 5% (4/83) for CDO and 10% (8/74) for KS. Due to the small numbers, this difference was not statistically significant (Fisher exact test [SISA], *p*~0.09); however, when the proportion of the affected chromosomes (rather than families) harboring *DNAI1 *mutation was compared, the difference between KS and CDO was statistically significant (*p*~0.008).

The prevalence of the IVS1+2-3insT mutation among the 22 mutated chromosomes was 50%, and that of A538T was 36%. To examine the possibility of founder effect(s) being responsible for their distribution in Polish population, *DNAI1 *mutations were analyzed in context of SNP haplotype background.

### SNP haplotype background

Variants of the 7-position SNP haplotype (flanked by markers located in exon 1 and intron 18 of the *DNAI1 *gene) were determined in 142 families, including 32 for which linkage of the disease phenotype with *DNAI1 *was excluded (all chromosomes from these 32 families were considered non-affected). Among the 142 families, 56 had both parents and one (15 families) or more (41 families) children genotyped, in 22 no genotype data was available for one or both parents, and in 64 only a singleton patient was genotyped. Converting the genotype data into haplotypes was aided by the fact that in almost half families (66 families, including 41 singleton patients) at least one of the members was homozygous or heterozygous only at a single SNP position, i.e., haplotype phase could be solved directly. Of the 142, 78 families were informative with respect to the parental contribution of the chromosomes. In the remaining families and in the multiply heterozygous singleton patients, determination of the alleles' phase from genotype data was based on the maximum parsimony principle, taking into account the frequency of the unambiguously determined haplotypes and assuming no recombination whenever possible. In three of the families, the unambiguous solution couldn't be achieved and these were excluded from further haplotype analysis.

**Figure 4:**
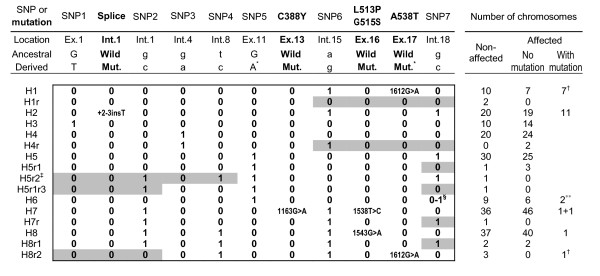
**Distribution of the 7-position SNP haplotype variants among the studied chromosomes**. Positions of the pathogenic mutations are indicated in on the SNP haplotype background. H1 through H8r2 are arbitrary names of the variants of the 7-position SNP haplotype. SNP1, 2, 3, 4, 5, 6 and 7 denote, respectively, rs11547035, rs4879792, rs2274591, rs3793472, rs11793196, rs9657620, rs11999046. Letters "0" and "1" in the left section of this Figure indicate, respectively, the ancestral and derived allele of the SNPs (the ancestral alleles were determined from the human-chimpanzee comparison, with the sequence identity indicating the ancestral state). Minimal regions of recombination (letter "r" in the haplotype name) in the rare haplotype variants, proposed assuming most parsimonious recombination among the frequent variants, and taking into account the extent of LD in the gene region (Figure 5), are highlighted. Right section of the Figure indicates the number of chromosomes with the respective haplotype variants. *G > A at rs11793196 and c.1612 are transitions at CpG dinucleotides. ^†^Mutation A538T on the H1 background was found only in KS families; A538T on the H8r2 was found in a CDO family. ^‡^Recombination detected within the PCD family. ^§ ^"0-1" at the last position of the H6 haplotype denotes ancestral allele (G) at rs11999046, linked with the derived allele (A) 93 nt downstream from rs11999046. **Unknown mutation(s) in two PCD families.

The resulting distribution of the haplotypes among 395 non-related chromosomes (183 non-affected and 212 affected) is given in Figure 4. Sixteen haplotype variants were distinguished. Their frequency did not differ significantly when the affected and non-affected chromosomes were compared. Eight of the haplotypes occurred at relatively high frequencies (4-21%) in the whole analyzed group of 395 chromosomes; the allelic structure of nine rare haplotypes (frequency ≤ 1%) suggested that they represented recent recombinants of the frequent variants. Only one of these recombination events was detected within the analyzed families; the remaining eight recombinants must have already "circulated" in the population.

The most prevalent IVS1+2-3insT mutation (found on eleven independent Polish chromosomes), was always found on the G-g-g-t-G-g-c haplotype (lower-case letters indicate SNPs in introns). This common background is consistent with the mutation's identity by descent (i.b.d.) in all the analyzed chromosomes. Another recurring mutation, A538T, was associated with the G-g-g-t-G-g-g haplotype on seven of the eight independent chromosomes, again indicating their recent common origin. Two chromosomes carrying unidentified mutations (in two unrelated patients with the monoallelic IVS1+2-3insT) had an identical haplotype G-g-g-t-A-a-g^a ^(g^a ^at the last position of the haplotype denotes ancestral "g" at rs11999046 linked with the derived "a" 93 nt downstream from rs11999046). The identity of the haplotypes background suggests that both families may share the same unidentified complementary mutation.

**Figure 5:**
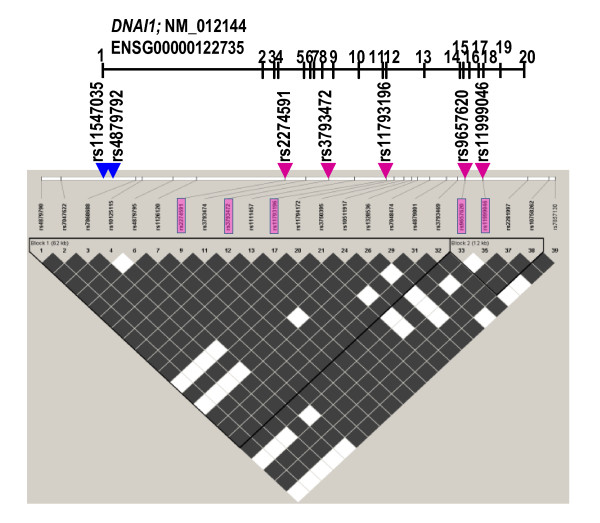
**Linkage disequilibrium (LD) across the *DNAI1 *gene**. Based on the HapMapCEU SNPs triangle plot generated for HapMap CEU data (release 21) for ENSG00000122735 (chrom 9:34457-34521 kb) with the use of Haploview software. Positions of SNPs genotyped in PCD families are indicated by arrowheads (rs11547035 and rs4879792, blue arrowheads, are not among SNPs from the HapMapCEU panel). The strength of the LD between SNPs (solid spine of LD) is indicated by colors: white (low) and dark (high); LD blocks are indicated by black triangles. Haplotypes flanked by markers rs11547036 and rs11793196 are within a single block of LD.

On the other hand, the background haplotypes for IVS1+2-3insT, A538T and the unknown mutation(s) are relatively frequent also among the non-affected chromosomes (10.9%, 5.5% and 4.9%, respectively). Therefore, the possibility that recurrent mutation events rather than i.b.d. are responsible for the relatively high frequency of these mutations cannot be excluded. In this context it has to be noted that the 1612G > A mutation on one of the eight chromosomes was associated with a different haplotype, G-g-g-c-G-g-g. The structure of the G-g-g-c-G-g-g cannot be explained by a single recombination event between two frequent haplotypes (Figure 4), and possible explanations include: 1) a double recombination or a gene conversion involving the frequent haplotype carrying the founder mutation (or its recombination with a very rare variant); 2) the possibility that the less common haplotype is derived from the common haplotype by a mutation at rs3793472; or 3) an independent mutation, which had occurred on a rare haplotype background. The first scenario would suggest the older age of the mutation, since the probability of a recombination or conversion event increases with time (e.g. [29]). Assuming the genomic average of 44 recombinations per meiosis per generation [30], the average genomic crossover rate is 10^-8 ^per bp per generation, and 6x10^-5 ^per 6 kb of the *DNAI1 *haplotype. One could therefore expect a single recombination event to occur once in 10^4 ^generations or once in ~200,000 years, and the probability of double recombination event is even lower. Moreover, the *DNAI *region flanked by markers rs11547036 (in exon 1) and rs11793196 (in exon 11), where at least one of the purported recombination events would have to take place, is characterized by the high level of linkage disequilibrium (Figure 5) in the HapMapCEU sample (http://hapmap.ncbi.nlm.nih.gov; [31,32]). The second scenario, of the recurrent mutation at rs3793472, would point to the identity of 1612G > A mutation in both haplotypes (G-g-g-t-G-g-g and G-g-g-c-G-g-g). However, since identical background haplotypes were also found on the unrelated healthy chromosomes (Figure 4), the t > c substitution at rs3793472 would have had to occur independently on the chromosomes with and without A538T mutation. With the average mutation rate of 1-4x10^-8 ^per bp per generation [33,34], this is not highly probable. Regarding the fact that the 1612G > A transition leading to A538T occurred within a CpG dinucleotide, known to mutate 10 times faster than other sequence positions [35], the third scenario, of an independent recurrent origin of this mutation, appears therefore most plausible.

## Discussion

### Prevalence of *DNAI1 *mutations among PCD families of various ethnicities

The disease-associated *DNAI *mutations were found in 8% of the analyzed Polish PCD families (12/157). This estimate is consistent with the previously reported *DNAI *involvement in 9% of PCD families (16/179) [18]; the earlier, even higher, reported values were based on much smaller study groups [13,16]. On the other hand, *DNAI1 *mutations were found in only 4% of the 104 PCD families analyzed in another study [20]. The authors suggested that the previously reported involvement of *DNAI1 *mutations reflected bias in the recruitment of PCD patients through detection of ODA defects. Indeed, the frequency of *DNAI1 *mutations in the pre-selected PCD subpopulation with documented ODA defects has been shown to be higher (14%) [2,18]. However, our estimate of 8% is based on the total number of Polish families, recruited without any preselection. Similarly, the criticized estimate of 9% [18] has been calculated with respect to all the 179 PCD families recruited for that study; even if the proportion of families with ODA defects in that cohort was shown to be ~80%, it did not necessarily reflect biased recruitment but rather the frequent presence of ODA defects among PCD patients in general. Therefore, the lowest reported involvement of *DNAI1 *[20] may reflect other factors, for example ethnic differences in the analyzed cohorts.

Of the 104 families analyzed by Failly et al. [20], 101 were Caucasian, with the predominant (3/4) contribution of Swiss (*n *= 50) or Italian (*n *= 32). The cohort analyzed by Zariwala et al. [18] was ethnically more heterogeneous: 155 of 179 families were Caucasian, and among 90 families for whom the ethnicity data were provided, the majority were German (*n *= 28), French (*n *= 23), UK (*n *= 18) and Australian (*n *= 11); only 6 samples were Italian, and no Swiss samples were reported. Our study group (*n *= 157) was predominantly Polish (*n *= 151); six families of Chech/Slovakian origin belong to the populations which are geographically and ethnically very close to Poles (all belong to West Slavs). Our results indicate that Poles (West Slavs), do not significantly differ from German, French or British populations when the *DNAI1 *involvement in PCD pathogenesis is considered [13,16,18]. The low prevalence of *DNAI1 *mutations among patients of Italian and Swiss origin [20] may either reflect specificity of these two populations or result from a clinal distribution of *DNAI1 *mutations, with the frequency gradient running in South-North rather than West-East direction. The existence of such gradients in Europe can be exemplified by the frequency distribution of the F508del mutation in the *CFTR *gene [36]. Answering the question whether the differences in *DNAI1 *involvement is due to the possible European clines in the geographical distribution of mutations or to the local founder effects will require studying PCD patients from other European populations.

### Population spectrum of *DNAI1 *mutations

The spectrum of *DNAI1 *mutations detected up to date in all the relevant studies is shown in Table 3. The prevalence of IVS1+2-3insT among the Polish PCD chromosomes harboring *DNAI1 *mutations (50%, 11/22) is only slightly lower than the respective value based on all the previous reports (56%, 27/48) [13,15,18,20]. The common background of this mutation in all the Polish chromosomes is consistent with their identity by descent. The origin of the recurring IVS1+2-3insT from the common founder has been suggested in earlier studies based on sharing allele 19CA of the nearby microsatellite D9S1805, located 0.26 Mb upstream of *DNAI1 *[18].

**Table 3:** The distribution of mutations reported up to date in the KS and CDO families

Study			Pennarun99		Guichard01		Zariwala01		Zariwala06		Failly08		This study		All	
			**KS**	**CDO**	**KS**	**CDO**	**KS**	**CDO**	**KS**	**CDO**	**KS**	**CDO**	**KS**	**CDO**	**KS**	**CDO**

			**Families (*n *= 487)**													

**Cohort size (# families)**			2	4	34	-	5	2	93	86	61	43	74	83	**269**	**218**

**Families with mutations detected** **(% of all studied PCD families)**		**2 mutations**		1	3		1	1	7	7	1	1	8	2	**21** **(8%)**	**17** **(8%)**

		**1 mutation**							1	1		2		2		

**Exon/Intron**	**Mutation**	**Effect**	**Chromosomes with mutations (*n *= 70)**													

In1	IVS1+2-3insT	S17fsX25		1	3		1	1	10	7	2	2	6	5	**22**	**16**

Ex5	282-283 insAATA	G95N fsX24		1												**1**

Ex6	463delA	T155LfsX18								1						**1**

Ex7	520G > A	E174L										1				**1**

In7	IVS7-2A > G	splicingAcc							1						**1**	

Ex10	874C > T	Q292X								1						**1**

In10	IVS10-4-7 delGTTT	splicingAcc								1						**1**

Ex13	1163G > A	C388Y											1		**1**	

"	1212T > G	Y404X							1						**1**	

"	1222G > A	V408M							1						**1**	

"	1307G > A	W436X								1						**1**

Ex16	1490G > A	R468-K523del								1						**1**

"	1538T > C	L513P											1		**1**	

"	1543G > A	G515S			2								1		**3**	

Ex17	1612G > A	A538T							1	1			7	1	**8**	**2**

"	1644G > A	W548X							1						**1**	

"	1657-68del	T553-F556del			1										**1**	

"	1703G > C	W568S					1					1			**1**	**1**

"	1704G > A	W568X						1								**1**

Ex19	1926-7insCC	I643PfsX48								1						**1**

In19	IVS19+1G > A	A607-K667del								1						**1**

**Number of chromosomes with *DNAI1 *mutations (% of all PCD chromosomes)**															**41 (8%)**	**29 (7%)**

Based on the data from [[13,15,16,18,20] this study].

The relatively high prevalence of A538T (36%, 8/22) appears to be specific for the Polish population; in all the other studies combined, this mutation represented only 1% (2/48) of *DNAI *mutations. Polish cohort is the only population where PCD patients homozygous for other alleles than IVS1+2-3T were found: three families without reported consanguinity were homozygous for A538T. The high frequency of A583T among Polish patients most likely reflects two phenomena: the common origin (founder mutation) in most families, and an independent mutation event on a different haplotype background in another family. Further studies involving other Eastern-European PCD cohort/s would be required to elucidate whether the founder mutation is restricted to the Polish population or characteristic for other Slavic groups. The excess of Polish PCD chromosomes harboring A538T was observed among the KS families; in fact, it is this mutation, which mostly contributed to the *DNAI1 *involvement being higher in KS than in CDO families.

Importantly for diagnostic purposes, A538T is located in exon 17, and two new mutations detected in this study (C388Y and L513P)-in exons 13 and 16, respectively, such that most of the mutant alleles remain clustered in intron 1 and exons 13, 16 and 17, as previously reported [2,18]. Chromosomes harboring mutations in these regions make up 80% of all the PCD chromosomes with the reported *DNAI1 *involvement. Of note, while the rare nonsense mutations or changes introducing a frame shift are distributed along the whole coding sequence, all but one (E174L) missense mutations are found in exons 13, 16 and 17.

### A question of unidentified *DNAI1 *mutations

Among a total of 38 PCD families with the *DNAI *mutations found in different studies, six were "monoallelic", with only one mutation identified in spite of direct sequencing of the whole coding region [[18,20] this study]. In four of these families, the single mutation found was the frequent IVS1+2-3insT. Is it possible that the affected members in these four families were just carriers of the detected mutation (with *DNAI1 *not being involved in PCD pathogenesis)? In such a case, the estimate of *DNAI1 *involvement in PCD pathogenesis would be slightly lower (7%; 34/487). With the disease prevalence of 1/20,000, *DNAI1 *involvement of ~7-10%, and IVS1+2-3insT prevalence among *DNAI1 *mutations of ~50%, the chance of picking up an asymptomatic IVS1+2-3insT carrier in the general population is ~1/535-1/450. Given that 487 independent PCD families were analyzed in all the reported studies, one would expect at most one of the patients to be an asymptomatic carrier of IVS1+2-3insT; the observed number of four carriers is higher, although the difference does not reach the level of statistical significance (*p *= 0.15; Fisher test). Nevertheless, we tentatively assume that *DNAI1 *is actually involved in PCD pathogenesis in the families with monoallelic mutation. In that case, the second mutation must have been undetectable by SSCP screening and direct sequencing of the amplified exonic segments. One of the possible explanations- the presence of long exonic deletion - was excluded, since the MLPA analysis using probes targeting all the *DNAI1 *exons did not reveal any differences in the amplification intensity of the PCD patients as compared to healthy controls. However, deep intronic or extragenic regulatory mutations remain to be searched for. Finally, the possibility that the inheritance of PCD in some families is di- or trigenic cannot be formally excluded, but so far no evidence exists which could substantiate this hypothesis.

## Conclusions

The analysis of the Polish PCD patients confirms large genetic heterogeneity of the disease and indicates that the worldwide involvement of *DNAI1 *mutations in PCD pathogenesis ranges from 7 to 10% in the families not preselected for the ODA defects; however, the involvement in specific populations may differ from this global estimate. In the combined PCD cohorts from all up to date studies, the IVS1+2-3insT remains the most prevalent pathogenetic change in *DNAI1 *(54% of all the mutations identified worldwide). The increased global prevalence of A538T (14%) is due to the contribution of the Polish cohort, in which the high frequency of this mutation (36%) probably reflects the local (Polish or Slavic) founder effect. The spectrum of mutations detected in the Polish cohort confirms earlier observations of mutations clustering in (or around) exons 1, 13, 16 and 17 of the *DNAI1 *gene, indicating directions for future diagnostic tests. Finally, with MLPA results indicating that no large exonic *DNAI1 *deletions are involved in PCD pathogenesis, the question of undetected mutations still remains open.

## Abbreviations

ASO: allele-specific oligonucleotide; CDO: ciliary dysfunction only; IDA: inner dynein arms; KS: Kartagener syndrome; MLPA: multiplex ligation-dependent probe amplification; MT: microtubules; ODA: outer dynein arms; PCD: primary ciliary dyskinesia; s.i.: situs inversus

## Competing interests

The authors declare that they have no competing interests.

## Authors' contributions

EZ designed and coordinated the study, performed haplotype analysis and interpretation of data, and drafted the manuscript, BN and KV carried the majority of SSCP and MLPA assays and participated in sequence analysis, US, ZB, KH and HP participated in SSCP assays and sequence analysis, ER was responsible for assembling, maintaining and monitoring the sample collection, AP recruited PCD families and provided clinical assessment of the patients, MW conceived the study and participated in its design. All authors read and approved the final manuscript.
